# Superficial Vein Thrombosis 21 cm Distant From the Venous Anastomosis of a Free Flap Causing Intermittent Venous Congestion

**Published:** 2009-07-30

**Authors:** Sammy Al-Benna, Hans-Ulrich Steinau, Lars Steinstraesser

**Affiliations:** Department of Plastic and Reconstructive Surgery, BG University Hospital Bergmannsheil, Ruhr University Bochum, Bochum, Nordrhein-Westfalen, Germany

## Abstract

**Objective:** Reconstructive surgeons are aware that deep vein thromboses can cause a free flap to fail; however, there are no reports in the literature regarding the incidence of free flap failure in the presence of superficial vein thromboses. **Methods:** A 38-year-old white male patient with poorly controlled type I diabetes mellitus presented with a chronically infected ulcer overlying an osteomyelitic right distal tibia. This region was reconstructed with a musculocutaneous latissimus dorsi free flap. **Results:** A superficial vein thrombosis was found 21 cm distant from the venous anastomosis of a free latissimus dorsi myocutaneous flap causing venous congestion. After early exploration, this flap was salvaged by resection of the compromised superficial venous system and the use of an interposition vein graft as no other suitable recipient vein was available. **Conclusions:** This case may serve as a reminder to explore early and be aware that the crux of a complication may be at a distance from the operative field. Consideration must be given to the possible need for interposition vein grafts when a suitable local deep or superficial recipient vein is not available in the lower limb.

Reconstruction of chronic osteomyelitic defects of the lower limb frequently requires free tissue transfer, and these often extensive bone and soft tissue debridements can occasionally be compounded by the presence of a deep venous thrombosis. There are no reports in the literature regarding the incidence of free flap failure in the presence of superficial vein thrombosis.

A 38-year-old white male patient with poorly controlled type I diabetes mellitus presented with a chronically infected ulcer overlying an osteomyelitic right distal tibia. Angiography revealed that only the posterior tibial artery was patent below the trifurcation of the popliteal artery.

## METHODS

Skin, soft tissue, and bone were extensively debrided, and 2 days later, the wound remained satisfactorily clean and, because of the extent of the soft tissue defect, it was elected to reconstruct this region with a musculocutaneous latissimus dorsi free flap.

**Figure 1:**
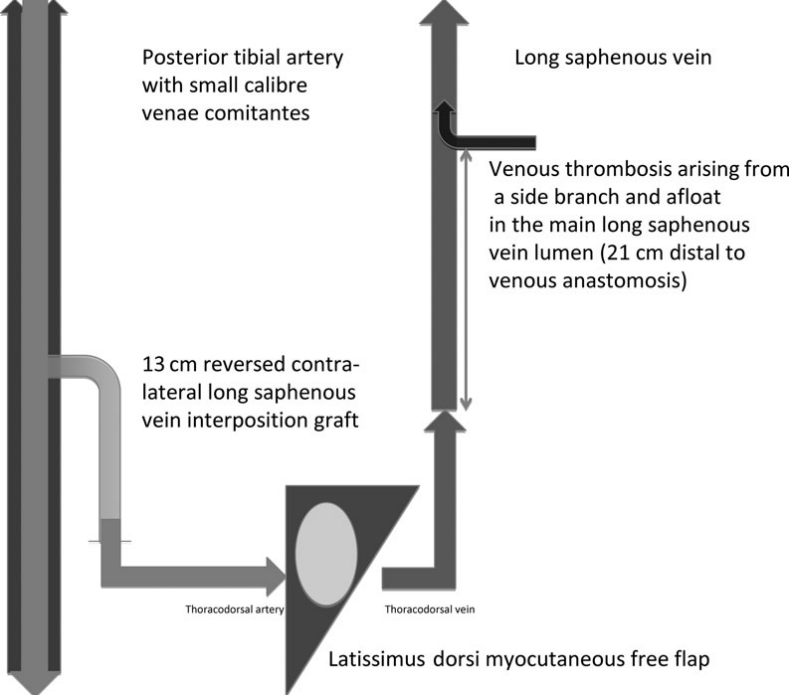
Vascular composition after the initial reconstruction.

The thoracodorsal artery of the flap was anastomosed end to side to the posterior tibial artery via a 13-cm length of reversed contralateral long saphenous vein interposition graft. Also, an end-to-end anastomosis between the thoracodorsal vein and the long saphenous vein (LSV) was performed as the diameter of the distal posterior tibial venae comitantes was inadequate (Fig 1). Systematic heparin was given intraoperatively, in addition to irrigation of the vessels. Operative time was 4 hours. Preoperatively and postoperatively, the patient received high-dose prophylactic low-molecular-weight heparin.

## RESULTS

The next 3 days were uncomplicated and the patient was at all times warm, well-perfused, and painfree. On the fourth postoperative day, venous congestion of the flap that intermittently resolved was observed several times over a 2-hour period. A decision was made to take the patient back to theatre. In the anaesthetic room, the flap again returned to a satisfactory state.

**Figure 2:**
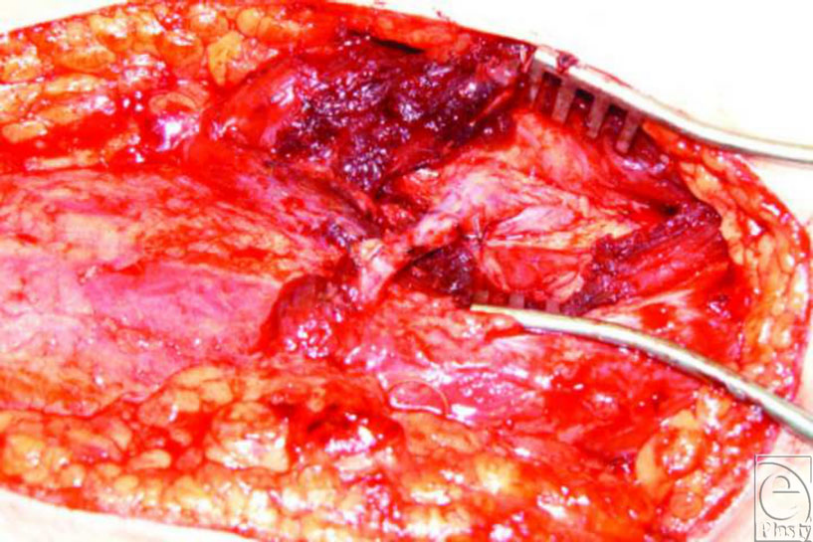
Dilated outflow long saphenous vein. The venous anastomosis was patent on exploration.

**Figure 3:**
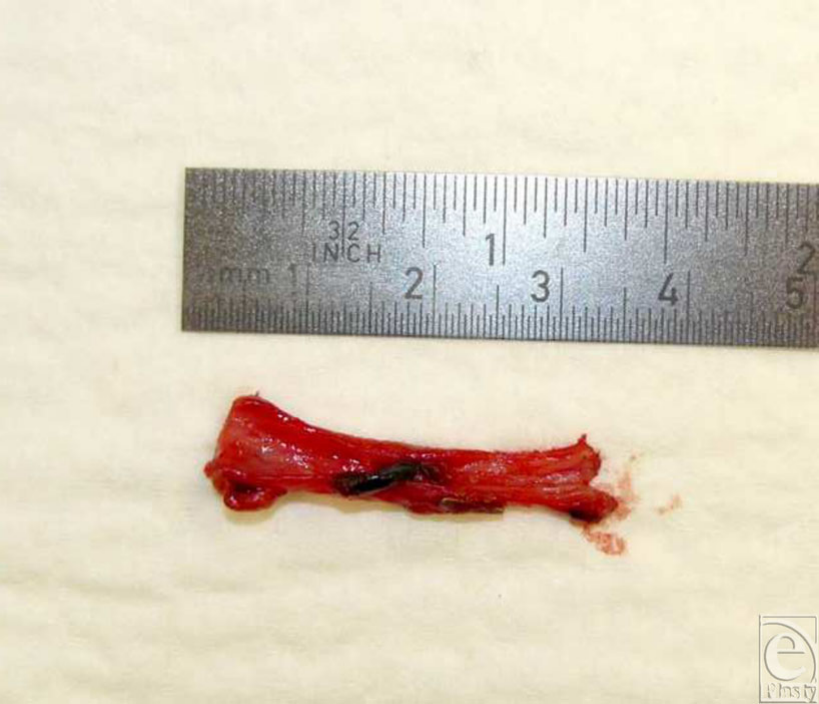
Thrombotic material in a side branch of the long saphenous vein propagating almost 1 cm into the main lumen. This was 21 cm distant to the venous anastomosis.

On exploration, the inflow arterial anastomoses and interposition vein graft were patent, while the outflow LSV was dilated and tense for a distance proximally (Fig 2). The venous anastomosis was patent; on following the dilated and tense LSV, thrombotic material was detected 21 cm proximal to the anastomosis in a side branch of the LSV propagating almost 1 cm into and afloat within the main LSV lumen (Fig 3). This explained the intermittent venous congestion of the flap. The vein and thrombus were resected and replaced with reversed contralateral LSV interposition graft. Postoperatively, the leg was elevated and the patient commenced on therapeutic low-molecular-weight heparin. No deep venous thrombosis was ever identified. There were no further postoperative complications, and the patient was discharged 14 days later.

## DISCUSSION

Early free flap complications include both arterial and venous thrombosis at the anastomotic site. Reconstructive surgeons are aware that deep vein thromboses can cause a free flap to fail; however, there are no reports in the literature regarding the incidence of free flap failure in the presence of superficial vein thromboses.

In this patient, intermittent flap failure occurred because of venous congestion from a superficial venous thrombosis over 20 cm distant to the anastomosis. This flap was salvaged because of early, thorough exploration, resection of the compromised superficial venous system, and the use of an interposition vein graft. There are a number of lessons to be learned from the experience of this case:

1. All flaps with signs of intermittent venous thrombosis should be investigated immediately.
2. The crux of a complication may be at a distance from the operative field.
3. Consideration must be given to the possible need for interposition vein grafts when a suitable local deep or superficial recipient vein is not available in the lower limb. Interposition vein grafts are clinically reliable and may be used without hesitation in appropriate clinical situations.^1^,^2^
4. Intraoperative and postoperative leg elevation may help with venous drainage.
5. Appropriate postoperative antithrombotic therapy should be considered.

Superficial vein thrombosis risk factors are close to those of venous thromboembolism.^3^–^6^ For superficial vein thrombosis of the lower limbs, which is the main location, varicose veins represent the principal cause, but underlying conditions and risk factors (including older age, male gender, trauma, immobilization longer than 3 days, major surgery, autoimmune diseases, malignancy, thrombophilia, corticosteroid use, chronic obstructive pulmonary disease, low albumin, and low hematocrit) must be sought in the absence of varicose veins.^3^–^6^ Concomitant deep vein thrombosis and pulmonary embolism can occur in approximately 15% and 5% of postoperative patients, respectively.^3^–^5^

Reconstructive surgeons need to salvage postoperative complications and this case may serve as a reminder to explore early and be aware that the crux of a complication may be at a distance from the operative field.
